# Early experience and future prospects regarding use of newly developed surgical robot system, hinotori, in the field of urologic cancer surgery

**DOI:** 10.1007/s10147-024-02503-5

**Published:** 2024-04-16

**Authors:** Hideaki Miyake, Masato Fujisawa

**Affiliations:** Division of Urology, Kobe University Graduate School of Medicine, 7-5-1 Kusunoki-Cho, Chuo-Ku, Kobe, 650-0017 Japan

**Keywords:** Hinotori, Robotic surgery, Urology

## Abstract

In the field of urology, robotic surgery has gained rapid and wide acceptance as a standard surgical approach in the majority of major surgeries over the last decade. To date, the da Vinci surgical system has been the dominant platform in robotic surgery; however, several newly developed robotic systems have recently been introduced in routine clinical practice. Of these, hinotori, the first made-in-Japan robotic system, is characterized by various unique and attractive features different from the existing system, and the use of this system has gradually increased mainly in urologic cancer surgeries, including radical prostatectomy, partial nephrectomy, radical nephrectomy, and radical nephroureterectomy. This review initially describes detailed characteristics of hinotori, then summarizes the early experience with urologic cancer surgeries using hinotori at our institution, and finally discusses the future prospects of robotic surgery using hinotori, considering problems associated with the use of this robotic system.

## Introduction

Revolutionary changes have occurred in minimally invasive surgery (MIS) following the recent introduction of robot-assisted surgery into real-world clinical practice. It has been well documented that the surgical robotic system is equipped with various advantageous features, such as three-dimensional (3D)-magnified clear vision, articulated instruments with multiple degrees of freedom, and scale motion for eliminating physiological tremors, which help overcome several limitations associated with purely laparoscopic approaches and efficaciously expand the indications of MIS through the use of surgical robotic systems in cases requiring highly complex procedures [1]. In the field of urology, robot-assisted surgery has been widely and promptly accepted as a standard approach for the majority of major surgeries, including robot-assisted radical prostatectomy (RARP), partial nephrectomy (RAPN), radical nephrectomy (RARN), radical nephroureterectomy (RANU), and radical cystectomy (RARC), and has generally shown findings superior to those of conventional open and laparoscopic surgeries [2, 3]

Over the last two decades, the da Vinci surgical system (Intuitive Surgical Inc., Sunnyvale, CA, USA) has dominated the market of surgical robot systems across the world; however, after the expiration of some relevant patents related to da Vinci, a number of novel robotic platforms have been under active development [4–8]. Among these, the hinotori surgical robot system, characterized by unique advantageous features different from existing platforms, was launched in 2019 by Medicaroid Corporation (Kobe, Japan) as the first made-in-Japan surgical robot system [8]. This system has already been used in several fields of robotic surgery, including urology, gynecology, and gastrointestinal surgery, and promising perioperative findings using hinotori have been reported [8–18].

In this review, the detailed process of hinotori development and its unique characteristics are initially described, and early experience with the use of hinotori for urologic cancer surgeries at our institution is then summarized. In addition, future prospects of robot-assisted surgery using hinotori are discussed based on problems with the use of this robotic platform.

## Development of hinotori

In 2013, Medicaroid Corporation was founded as a joint venture of Kawasaki Heavy Industries, Ltd. (Kobe, Japan) and Sysmex Corporation (Kobe, Japan) to expand their advanced robotic technology to the medical field and started the development of a novel surgical robot system in 2015. A total of five prototypes of the robotic platform were produced to optimize the mechanical movement as well as software control up until 2019. In 2020, the surgical robot for clinical use was completed, and was named hinotori, based on the story that the feathers of the phoenix heal all injuries and diseases in “hinotori,” one of the masterpieces by a famous Japanese cartoon artist, Osamu Tezuka.

In 2020, hinotori obtained Japanese regulatory approval in August and insurance coverage in September, and RARP as the first surgery using hinotori was performed in December at the International Clinical Cancer Research Center of Kobe University Hospital (Kobe, Japan) [8].

## Characteristics of hinotori

**Fig. 1:**
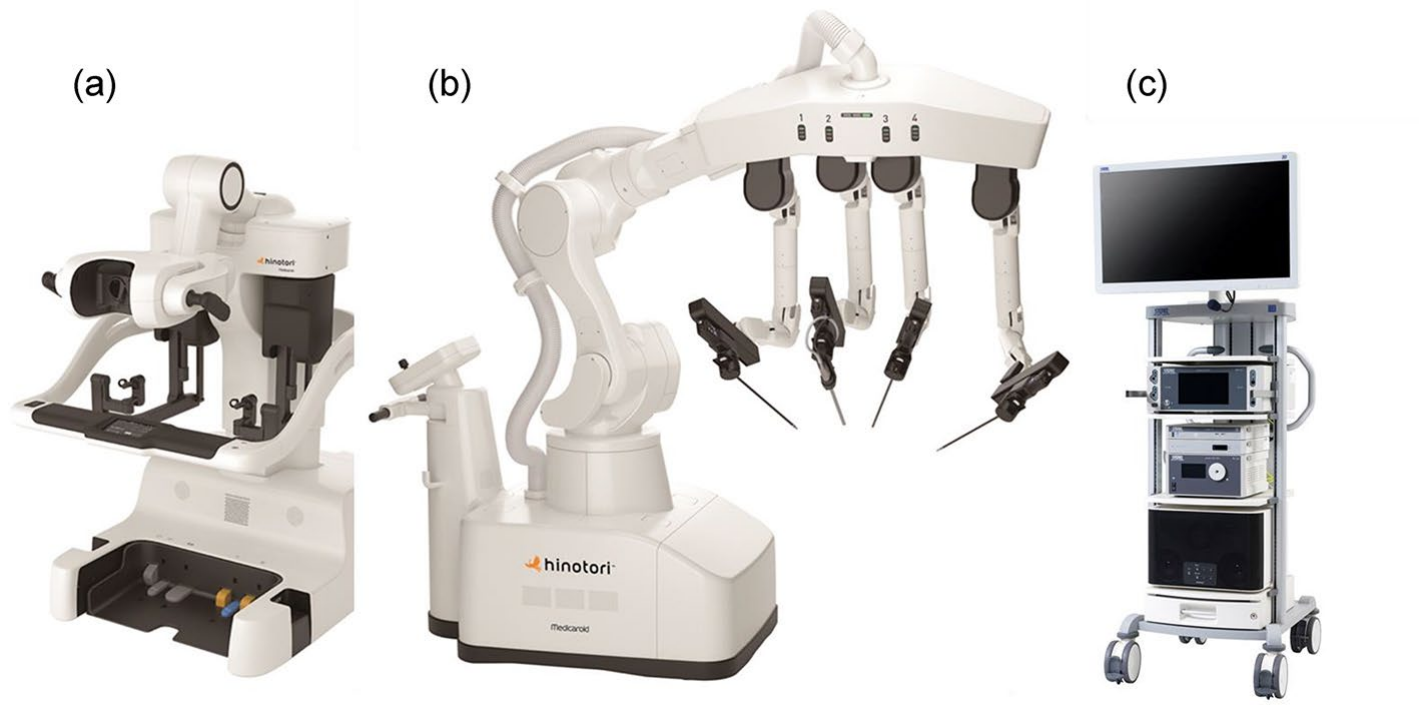
The hinotori surgical robot system (Medicaroid Corporation, Kobe, Japan), consisting of (**a**) a surgical cockpit and (**b**) an operation unit, which are ergonomically designed to reduce the burden on the surgeon, with (**c**) a monitor cart displaying a high-definition three-dimensional endoscopic image

The hinotori surgical robot system is composed of three units, like the da Vinci system, including a surgical cockpit, an operation unit, and a monitor cart (Fig. 1). However, it has several unique features different from da Vinci as follows: (1) compact robotic arms with eight axes of motion, one more than the da Vinci system, are mounted in hinotori and are controlled by a computer system to realize more flexible movement and minimize interference among the arms. (2) The trocar position is calibrated by software without docking of an arm with a port, which helps provide a sufficient working space in a clean field and protect against collisions among arms outside the body. (3) A flexibly positioned full high-vision 3D viewer mounted in the surgical cockpit, which provides a 16:9 wide-view high-definition image, which makes it possible to perform accurate surgery and reduce the fatigue of surgeons. These characteristics could create a suitable environment for urologic cancer surgery, particularly that requiring highly complex procedures.

Instruments available during robotic surgery using hinotori include bipolar fenestrated forceps, bipolar Maryland forceps, monopolar curved scissors, standard and wide needle holders, three types of grasping forceps, and clip appliers for L-, ML-, and S-size clips.

## Preclinical study

Before introducing hinotori into clinical practice, preclinical studies were elaborately performed, and detailed findings from these studies were previously described [8]. Briefly, an initial preclinical study was conducted using ten living female pigs under general anesthesia at the Medical Device Innovation Platform (Kobe, Japan). Four surgical procedures, right RAPN, left RAPN, vesicourethral anastomosis, and pelvic lymphadenectomy, per pig were performed by five surgeons with experience of > 300 robot-assisted surgeries. All procedures were completed, and the mean operative time was 47.6, 13.9, and 34.8 min for RAPN, vesicourethral anastomosis, and pelvic lymphadenectomy, respectively. Out of a total of 40 procedures, 4 (10%) recoverable errors occurred due to arm collisions during RAPN.

Cadaveric studies were performed using four male cadavers at the Clinical Anatomy Training Center in Kobe University Graduate School of Medicine (Kobe, Japan). Right RAPN, left RAPN, RARP, and pelvic lymphadenectomy per each cadaver were performed by three surgeons with experience of > 300 robot-assisted surgeries. All 16 procedures were successfully completed, and the mean operative time was 57.0, 63.7, and 70.3 min for RAPN, RARP, and pelvic lymphadenectomy, respectively. Recoverable arm collision occurred during two procedures, RAPN and RARP.

## RARP using hinotori

After obtaining Japanese regulatory approval, a multi-institutional observational study of RARP using hinotori was conducted as a first-in-human clinical study at four Japanese institutions, including ours. This study consisted of a total of 30 patients with clinically localized or locally advanced prostate cancer (clinical stage T1–T3a, N0, M0) and employed six surgeons with certification as a proctor for robot-assisted surgery by the Japanese Society of Endourology and Robotics.

All RARPs were performed according to the same method as previously reported [19] and were successfully completed. In this series, the median time using hinotori and estimated blood loss were 165 min and 162.5 mL, respectively, and four (13.3%) recoverable errors occurred. Adverse events corresponding to Clavien–Dindo classification ≥ 3 were observed in three (10%) cases and positive margins were detected in four (13.3%) cases. These perioperative findings were comparable with those in a previous report of the 30 initial cases of RARP using da Vinci [20]. Although the number of patients included in this study was insufficient, and additional assessments on other important issues will be required [21, 22], it is significant to show comparatively favorable findings for RARP, the most prevalent robotic surgery in the field of urology, using hinotori.

## RAPN using hinotori

After the promising outcomes were documented in the first-in-human clinical study of RARP, the use of hinotori has expanded to several types of surgeries. Of these, RAPN may be one of the most suitable surgeries for the use of hinotori considering the several unique characteristics described above, which may facilitate performing RAPN, particularly for patients with technically challenging, complicated tumors.

After the application of hinotori to RAPN at our institution, the majority of RAPNs were conducted by hinotori, and the initial experience with RAPN using hinotori was investigated focusing on perioperative findings in 30 prospectively included patients with small renal tumors [9]. Surgical procedures of RAPN using hinotori in this study were identical to those using da Vinci, which were previously described [23, 24]. In this first series, RAPN using hinotori could be successfully completed in all the 30 patients as preoperatively planned, and favorable perioperative outcomes could be achieved: the median operative time, time using hinotori, and warm ischemia time were 179, 106, and 13 min, respectively, while a positive surgical margin was not detected, and major perioperative complications did not occur in these 30 patients. Accordingly, trifecta outcomes, the most commonly used surrogate showing successfully performed partial nephrectomy, were achieved in all the 30 patients. These perioperative outcomes, except for the postoperative hospital stay, were comparable to those of RAPN in previous studies from high-volume centers [25, 26].

Following the first report on RAPN using hinotori, perioperative findings on RAPN using da Vinci Xi and hinotori were compared [10]. In this study, 303 and 40 patients undergoing RAPN using da Vinci Xi and hinotori, respectively, were included, and potential baseline parameters were adjusted by propensity score matching, resulting in the generation of 2 cohorts, consisting of 74 and 37 undergoing RAPN using da Vinci Xi and hinotori, respectively. As summarized in Table 1, no significant differences in major perioperative outcomes were noted between the da Vinci Xi and hinotori groups. Collectively, these findings indicate that RAPN using hinotori could provide non-inferior perioperative outcomes compared with those using the existing system, da Vinci Xi; however, it is necessary to further compare important issues, such as prognostic outcomes, renal function, quality of life, and these in patients with complicated tumors [27, 28], between these two robotic platforms.

**Table 1:** Comparison of perioperative outcomes between hinotori and da Vinci Xi groups after propensity score matching

	hinotori group (*n* = 37)	da Vinci Xi group (*n* = 74)	*P* value
Operative time (min)	171	171	0.54
Time using robotic system (min)	108	107	0.79
Warm ischemia time (min)	12	12	0.30
Estimated blood loss (mL)	34	50	0.20
Positive cancer margins (%)	0 (0)	0 (0)	1.00
Postoperative complications (%)*	0 (0)	0 (0)	1.00
Achievement of trifecta (%)	100 (0)	100 (0)	1.00
Reduction in eGFR (%)	9.2	8.9	0.83

*eGFR* estimated glomerular filtration rate

*Corresponding to Clavien–Dindo 3 or 4

## RARN using hinotori

Since the initial report in 2005 by Klinger et al. [29], the proportion of renal cell carcinoma patients undergoing RARN has markedly increased, and RARN has been regarded as a promising alternative to laparoscopic radical nephrectomy [30, 31]. We also recently reported the first experience with RARN using da Vinci Xi in Japan and showed favorable perioperative findings [32]. Consistent with the use of da Vinci Xi for RARN, RARN using hinotori was initiated, and perioperative outcomes of 13 patients with renal cell carcinoma managed by RARN using hinotori were initially described [11]. In these 13 patients, RARN could be successfully completed as preoperatively planned using hinotori without transfusion or conversion to open surgery, and favorable perioperative outcomes could be achieved in these 13 as follows: operative time, 157 min time using hinotori, 83 min; estimated blood loss, 11 mL; and no major complications, which were similar to those of our initial series of RARN using da Vinci Xi [32].

**Fig. 2:**
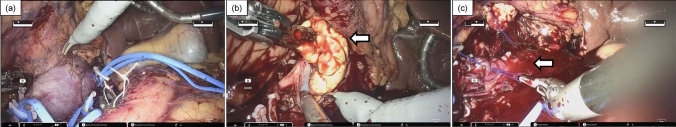
Intraoperative images during robot-assisted radical nephrectomy and inferior vena cava (IVC) tumor thrombectomy for a patient with renal cell carcinoma and a level II IVC tumor thrombus. **a** The left renal vein, caudal IVC, and cephalic IVC were clamped with twice-wrapped vessel loops by clipping in addition to the use of bulldogs. **b** The tumor thrombus (arrow) was removed from IVC, and the wall of IVC was cut. **c** IVC was reconstructed by continuous suture with 4–0 polypropylene (arrow), after removing the tumor thrombus

In recent years, the application of RARN has expanded to patients with renal cell carcinoma with an inferior vena cava (IVC) tumor thrombus, and the outcomes of RARN and IVC tumor thrombectomy have been shown to be superior to those of conventional open surgery with respect to blood loss and perioperative complications [33, 34]. We also first applied a purely robotic approach using da Vinci Xi to the treatment of a patient diagnosed with renal cell carcinoma with an IVC thrombus in Japan in 2021 [35] and are now continuously performing this approach for these patients. Furthermore, to date, two patients with renal cell carcinoma with an IVC thrombus have been treated with a purely robotic approach using hinotori [13]. Both operations were conducted with the use of hinotori based on procedures that are the same as those in cases using da Vinci Xi [35] and were successfully completed without any major perioperative complications, resulting in the following findings: operative time, 228 and 214 min; time using hinotori, 158 and 156 min; and blood loss, 535 and 200 mL, respectively (Fig. 2). These findings were comparable to those with da Vinci Xi in our cases; accordingly, purely robotic surgery using hinotori may be an effective and safe treatment for renal cell carcinoma patients with an IVC tumor thrombus.

## RANU using hinotori

**Fig. 3:**
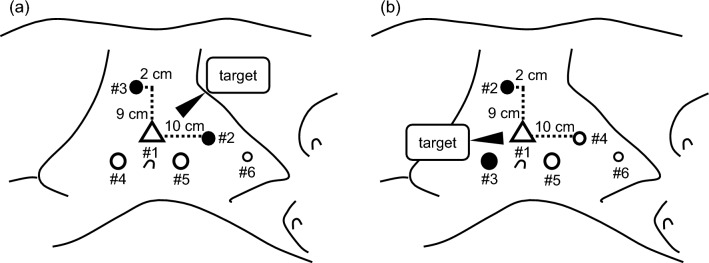
Trocar placement in cases of robot-assisted right radical nephroureterectomy using hinotori. **a** Trocar placement at the kidney direction stage. #1, a 12-mm camera port; #2, an 8-mm robotic port for the right arm; #3, an 8-mm robotic port for the left arm; #4, a 12-mm assistant port; #5, a 12-mm assistant port; #6, a 5-mm assistant port for liver traction. **b** Trocar placement at the bladder direction stage. #1, a 12-mm camera port; #2, an 8-mm robotic port for the right arm; #3, an 8-mm robotic port placed inside the 12-mm port for the left arm; #4, an 8-mm assistant port; #5, a 12-mm assistant port; #6, a 5-mm assistant port (not used during this stage)

Since the initial report in 2006 [36] and the subsequent studies presenting technical refinements [37, 38], RANU has been regarded as one of the standard surgical options for patients with upper urinary tract cancer in routine clinical practice, and recent studies have clarified several advantages of RANU over other approaches, including open and laparoscopic surgeries [39, 40]. At our institution, following the introduction of RANU using da Vinci Xi, RANU with the use of hinotori was started, and after accumulating experience of RANU using hinotori for eight patients, initial experience of this surgery was reported [12]. In this series, RANU, including bladder cuff excision and lymph node dissection, was completed in all the eight patients as preoperatively scheduled. During RANU in these eight patients, reconfiguration of robotic arms from the kidney to bladder direction stage made it possible to precisely manage the upper abdominal as well as deep pelvic spaces (Fig. 3); therefore, all surgical procedures were accomplished without repositioning of the patient or port. Furthermore, the following favorable perioperative findings were achieved: operative time, time using hinotori, and blood loss were 230 min, 138 min, and 23 mL, respectively, and major perioperative complications were not observed. Despite being obtained from a small case series, these outcomes suggest that, considering the efficacious and safe perioperative findings, RANU using hinotori may be a useful alternative to open and laparoscopic approaches, being similar to using the existing platform, da Vinci.

## Problems and future prospects with the use of hinotori

Since the first-in-human surgery at the end of 2020, the number of robotic surgeries using hinotori, mainly urologic cancer surgery, has been gradually increasing. Up until 2023, a total of 2314 patients with urological cancers underwent robotic surgeries with the use of hinotori, including 1893, 266, 83, 56, and 16 receiving RARP, RAPN, RARN, RANU, and RARC, respectively. To date, however, several problems associated with the use of hinotori, which should be promptly overcome, have been pointed out. For example, the lineup of currently available instruments is insufficient to perform some types of robotic surgeries, which may explain the low number of patients undergoing RARC. In addition, hinotori has no annotation function; thus, quality of mentoring during robotic surgery using hinotori is not high enough. If these problems can be resolved, the indications of hinotori will be further expanded to more complex robotic surgeries.

On the contrary, there are a number of projects related to hinotori in progress. Of these, the following issues may be particularly important to expand the specialties of hinotori, including cardiovascular surgery and thoracic surgery, along with currently involved specialties (urology, gynecology, and gastrointestinal surgery): expansion of instrument lineup, enhancements of imaging systems and electrosurgical units, and developments of intraoperative navigation systems and synchronized beds. Furthermore, projects for further advancement of the field of robot-assisted surgery are also underway, such as remote surgery, robotic autonomy, and artificial intelligence analysis of surgical procedures. In parallel with such refinements associated with robotic equipment, global business regarding hinotori is scheduled to be expanded across the world.

## Conclusion

The first made-in-Japan robotic system, hinotori, is characterized by several unique features different from the existing system, which help perform urologic cancer surgery, particularly that requiring highly precise procedures for complex cases. In real-world clinical practice, the use of hinotori for urologic cancer surgery has been gradually increasing, and there have been several studies reporting perioperative findings with these surgeries, including RARP, RAPN, RARN, and RANU, comparable with those using the existing system. Furthermore, numerous projects are in progress for further refinement of robotic surgery using hinotori. Collectively, these findings suggest the future expansion of robotic surgery with the use of hinotori to a wide variety of specialties.
